# Preliminary Investigation on the Ameliorative Role Exerted by D-Aspartic Acid in Counteracting Ethane Dimethane Sulfonate (EDS) Toxicity in the Rat Testis †

**DOI:** 10.3390/ani11010133

**Published:** 2021-01-08

**Authors:** Massimo Venditti, Maria Zelinda Romano, Francesco Aniello, Sergio Minucci

**Affiliations:** 1 Dipartimento di Medicina Sperimentale, Sez. Fisiologia Umana e Funzioni Biologiche Integrate “F. Bottazzi”, Università della Campania “Luigi Vanvitelli”, Via Costantinopoli, 16, 80138 Napoli, Italy; massimo.venditti@unicampania.it (M.V.); mariazelinda.romano@unicampania.it (M.Z.R.); 2 Dipartimento di Biologia, Università di Napoli ‘Federico II, Via Cinthia’, 21, 80126 Napoli, Italy; faniello@unina.it

**Keywords:** D-aspartic acid, EDS, testosterone, testis, StAR, PREP, endocrine disrupters

## Abstract

**Simple Summary:**

For proper fertility, the production of good-quality spermatozoa is essential. Nowadays, many environmental pollutants affect the spermatogenetic process, at different levels. For this reason, new approaches are needed to prevent/counteract these toxic effects. Here, we showed that the excitatory amino acid D-aspartic acid (D-Asp) prevents the deadly action of ethane dimethane sulfonate (EDS) on the testosterone-secreting Leydig cells in rat testis. We found that EDS, probably via the reduced testosterone level, alters the normal histology of the seminiferous epithelium, leading to germ cells death and to the decreased protein level of two Leydig cell “markers”: steroidogenic acute regulatory and prolyl endopeptidase. In addition, the same analysis performed on rats that were pre-treated with D-Asp revealed a protective role of this compound, since all the above parameters were quite normal. Moreover, we found that the protective mechanism of action involved in this scenario may be due to the ability of D-Asp to reduce the oxidative stress induced by EDS. Based on these findings, we could affirm that D-Asp may be an encouraging candidate to be used to alleviate the harmful action due to environmental pollutants exposure, in order to maintain appropriate fertility.

**Abstract:**

Herein is reported the first evidence of the protective role of D-aspartic acid (D-Asp) in preventing the toxic effect exerted by the alkylating agent ethane dimethane sulfonate (EDS) in the rat testis. We confirmed that EDS treatment specifically destroyed Leydig cells (LC), resulting in the drastic decrease of the serum testosterone level and producing morphological changes in the germinal tubules, i.e., altered organization of the epithelium, loss of cell contacts and the consequent presence of empty spaces between them, and a reduce number of spermatozoa. Moreover, an increase of TUNEL-positive germ cells, other than alteration in the protein level and localization of two LC “markers”, StAR and PREP, were observed. Interestingly, results obtained from rats pre-treated with D-Asp for 15 days before EDS-injection showed that all the considered parameters were quite normal. To explore the probable mechanism(s) involved in the protection exerted by D-Asp, we considered the increased oxidative stress induced by EDS and the D-Asp antioxidant effects. Thiobarbiturc acid-reactive species (TBARS) levels increased following EDS-injection, while no change was observed in the D-Asp + EDS treated rats. Our results showed that D-Asp may be used as a strategy to mitigate the toxic effects exerted by environmental pollutants, as endocrine disrupters, in order to preserve the reproductive function.

## 1. Introduction

Male germ cells production is a finely regulated process which occurs in the extensive seminiferous epithelium, which is composed of two cell types: germ cells in different stages of differentiation, and Sertoli cells, which support and drive the germ cell during their differentiative progression [1]. Between the tubules, there is a connective tissue, constituted of blood and lymphatic vessels, nerve fibers, fibroblasts, scattered mast cells, macrophages, and the testosterone (T)-secreting Leydig cells (LC) [2]. Spermatogenesis can be divided into three phases: mitotic division of spermatogonia, meiosis to form round spermatids and, finally, their differentiation into mature spermatozoa via the spermiogenesis. All the steps not only are under genetic control, since specific genes are coordinately activated, [3,4,5,6], but also under hormonal control, via the hypothalamus-hypophysis-testis (HPT) axis. Besides the well-known regulative action exerted by the canonical modulators of gametogenesis, such as gonadotropins, T and retinoic acid [7,8], an intricate network, and endocrine, paracrine, and autocrine elements act both centrally and locally, governing all the steps of spermatogenesis with the fundamental objective to produce good quality spermatozoa, that will ensure the reproductive success [9,10,11,12].

D-aspartic acid (D-Asp) is one of the most studied factors in this regard. This D-amino acid is widely distributed in mammalian nervous and endocrine systems [13,14] controlling male gametogenesis thanks to its regulative role on two different levels. Indeed, D-Asp acts on the HPT axis, stimulating GnRH [15], luteinizing hormone (LH), and T [16] synthesis and release, as well as on the testicular cells [17,18,19,20], eliciting molecular pathways involved in LC steroidogenesis and in spermatogonia proliferation [21]. Moreover, we recently demonstrated that D-Asp is also involved in cytoskeletal remodeling, one of the main aspects of spermatogenesis [22,23], since it regulates the protein levels of prolyl endopeptidase (PREP) [24] and disheveled-associated activator of morphogenesis 1 (DAAM1) [25], that are associated to microtubules and actin, respectively. In particular, as PREP expression increased particularly in the rat LC cytoplasm following D-Asp acute (a single i.p. injection with 2.0 µmol/g body weight D-Asp dissolved in a saline solution) and chronic (20 mM of D-Asp in drinking water for 15 days) treatment, we hypothesize the involvement of this serine protease in controlling the sexual hormone homeostasis, essential for spermatogenesis [24].

Given this background, the potentialities of D-Asp are evident, but far from being completely understood. Indeed, although in the scientific literature there are several studies concerning the role of D-Asp in the male gametogenesis in “physiological” condition, there are just few papers on its involvement in pathological circumstances, such as infertility [26,27,28], or with the aim to evaluate its possible protective action against reproductive toxicants and endocrine disrupters. For this reason, the scope of this work is to evaluate whether D-Asp may have a role in preventing the hormonal imbalance caused by several chemical compounds. We used the ethane dimethane sulfonate (EDS), an alkylating agent that selectively destroys LC in the interstitial compartment of many vertebrates [29,30,31,32,33,34,35,36,37,38]. This compound is widely used in research not only focused on the examination of LC physiology and their regenerative features since, following about 7 days after the treatment, these cells can regenerate, but also to verify the effects of the lack of T in the testicular environment lacking T. In this study, we used rats treated with D-Asp prior EDS injection, to evaluate the T levels, the testicular histology, the apoptotic rate of interstitial and tubular cells, some LC markers other than an oxidative stress parameter.

## 2. Materials and Methods

### 2.1. Reagent and Antibodies

Reagents and antibodies were purchased from the following sources: (1) D-Asp (219096; Sigma-Aldrich, Milan, Italy); (2) DMSO (D8418; Sigma-Aldrich); (3) chloral hydrate (47335-U; Sigma-Aldrich); (4) T ELISA kit (#ab108666; Abcam, Cambridge, UK); (5) DeadEnd™ Fluorometric TUNEL System (G3250; Promega Corp., Madison, WI, USA); (6) Vectashield + DAPI (H-1200-10; Vector Laboratories, Peterborough, UK); (7) 2-thiobarbituric acid (T5500; Sigma-Aldrich); (8) SDS (L3771; Sigma-Aldrich); (9) NP-40 (492016; Sigma-Aldrich); (10) sodium orthovanadate (S6508; Sigma-Aldrich); (11) sodium deoxycholate (D6750; Sigma-Aldrich); (12) leupeptin (L2884; Sigma-Aldrich); (13) chymostatin (C7268; Sigma-Aldrich); (14) aprotinin (A6103; Sigma-Aldrich); (15) pepstatin A (P5318; Sigma-Aldrich); (16) PMSF (P7626; Sigma-Aldrich); (17) PVDF membranes (GE10600023; Amersham Pharmacia Biotech, Buckinghamshire, UK); (18) Tween-20 (P9416; Sigma-Aldrich); (19) skim milk powder (M7409; Sigma-Aldrich); (20) Triton-X 100 (T8787; Sigma-Aldrich); (21) BSA (05470; Sigma-Aldrich); (22) normal goat serum (NS02L; Sigma-Aldrich); (23) anti-StAR antibody (E-AB-15419, Elabscience Biotechnology, Wuhan, China); (24) anti-PREP antibody (ab58988; Abcam); (24) anti-α-tubulin antibody (E-AB-20036; Elabscience Biotechnology); (25) horseradish peroxidase-conjugated anti-mouse IgG (AP130P; Sigma-Aldrich); (26) horseradish peroxidase-conjugated anti-rabbit IgG (AP307P; Sigma-Aldrich); anti-PCNA antibody (#sc-56; Santa Cruz Biotechnology, Inc., Dallas, TX, USA); (27) anti-rabbit Alexa Fluor 488 (A32731; Thermo Fisher Scientific, Waltham, Ma, USA); (28) anti-mouse IgG 568 (SAB4600082; Sigma-Aldrich).

### 2.2. Animals and Experiments

Male adult Wistar rats (*Rattus norvegicus*), weighing 300 to 350 g, were kept under regulated temperature (24 ± 2 °C) and lighting conditions (12 h light and 12 h dark cycles). They received commercial food pellets ad libitum. Rats (*n* = 15) were divided into three groups: the first group (*n* = 5) was allowed to drink a solution consisting of 20 mM D-Asp for 15 days [39]; to the second group of rats (*n* = 5), fresh water for 15 days was given. At the end of the above period, both groups were injected intraperitoneally with EDS (100 mg/kg body wt in DMSO–dH_2_O 1:3 v/v; 200 μL/animal). The third group (*n* = 5) was exposed neither to D-Asp or EDS and was used as a control. After 5 days from the EDS injection, in the early daylight hours, rats were first anesthetized by an intraperitoneal injection of chloral hydrate and then sacrificed. For each animal, the testes were immediately dissected out: the left was rapidly immersed in Bouin’s fluid while the right in liquid nitrogen for histochemical and biochemical analysis, respectively. The experimental protocol and the housing conditions were in accordance with the Italian guidelines (D. Lvo 116/92) and authorized by the local Animal Care Committee (Servizio veterinario ASL 44, Prot. Vet. 22/95).

### 2.3. Histology

The fixed testes were dehydrated in increasing alcohol concentrations before paraffin embedding. Five-μm thick serial sections were stained with hematoxylin/eosin. For histopathological evaluation, 20 seminiferous tubules/animal for a total of 100 tubules per group were counted under microscope (Leica DM 2500, Leica Microsystems, Wetzlar, Germany). Photographs were taken using the Leica DFC320 R2 digital Camera.

### 2.4. Serum Testosterone Evaluation

For the assessment of serum testosterone concentration, after euthanasia, blood samples were collected by cardiac puncture in sterile and heparinized tubes and then centrifuged at a speed of 5000× *g* for 10 min at +4 °C. Each sample was analyzed by ELISA method using a commercial kit.

### 2.5. TUNEL Assay

Apoptosis was examined in paraffin sections by the TUNEL-assay using DeadEnd™ Fluorometric TUNEL System following manufacture’s protocol. Briefly, sections were dewaxed, rehydrated, and washed in 0.85% NaCl and then PBS (13.6 mM NaCl; 2.68 mM KCl; 8.08 mM Na_2_HPO_4_; 18.4 mM KH_2_PO_4_; 0.9 mM CaCl_2_; 0.5 mM MgCl_2_; pH 7.4). To permeabilize the tissues, slides were treated with proteinase K for 10 min at RT. Then, slides were equilibrated in a specific buffer for 10 min at RT, followed by incubation with TdT enzyme and nucleotide mix for 1 h at 37 °C. Reaction was stopped with incubation for 10 min in SSC2X. Finally, the cells nuclei were marked with Vectashield + DAPI. The sections were observed and captured with the optical microscope (Leica DM 5000 B + CTR 5000) with UV lamp and saved with IM 1000 software.

### 2.6. Thiobarbiturc Acid-Reactive Species (TBARS) Levels Assessment

Testis lysate were incubated with 0.5 mL of 0.78% aqueous solution of thiobarbituric acid 0.5 mL of 20% acetic acid, pH 3.5. Samples were heated for 45 min at 95 °C and then centrifuged at 4000× *g*. for 5 min. Supernatant were collected and TBARS were quantified by spectrophotometry at 532 nm [40]. Results were expressed as TBARS µM/µg of extracted protein. Each measurement was performed in triplicate.

### 2.7. Protein Extraction and Western Blotting Analysis

Testes were lysed in RIPA lysis buffer [0.1% SDS, 1% NP-40, 100 mM sodium orthovanadate, 0.5% sodium deoxycholate, in PBS supplemented with protease inhibitors (4 μg/μL leupeptin, chymostatin, aprotinin, pepstatin A, and PMSF)]. The homogenized samples were sonicated 3 times (20 Hz for 20 s each), placed on ice for 30 min, centrifuged at 10,000× *g* for 30 min at 4 °C; finally, the resulting supernatants were collected. Forty micrograms of the protein extracts were separated by 9% SDS–PAGE and transferred to Hybond-P polyvinylidene difluoride membranes at 280 mA for 2.5 h at 4 °C. Filters were blocked with 5% skim milk in TBST (10 mM Tris–HCl pH 7.6, 150 mM NaCl, containing 0.25% Tween-20) for 3 h at RT. Then, they were incubated with primary antibodies overnight at 4 °C: (1) anti-StAR (1:500); (2) anti-PREP (1:3000); and (3) anti-α-tubulin (1:5000) diluted in the blocking solution After washing thrice with TBS/Tween, the membranes were incubated with horseradish peroxidase-conjugated secondary antibody anti-mouse IgG for the mouse anti-α-tubulin or anti-rabbit IgG secondary antibody for the other two, both diluted 1:10,000 in the blocking mixture for 1 h at room temperature. Then, membranes were washed again thrice in TBS/Tween and the immunocomplexes were detected using the enhanced chemiluminescence (ECL)-Western blotting detection system. ImageJ software (version 1.53g) was used to analyze all bands. Western blotting was performed in triplicate.

### 2.8. Immunofluorescence Analysis

For immunofluorescence staining, testis sections were permeabilized with PBS pH 7.4 containing 0.1% Triton-X-100 for 30 min after deparaffinization and rehydration. Antigen retrieval was performed by pressure cooking slides for 3 min in 0.01 M citrate buffer (pH 6.0). Then, non-specific binding sites were blocked with PBS containing 5% BSA and normal goat serum diluted 1:5. Later, sections were incubated with anti-StAR, anti-PCNA, anti-PREP, and anti-α-tubulin antibodies (all diluted 1:100 in the blocking solution) overnight at 4 °C. After three washes in PSB, the appropriate secondary antibody diluted 1:500 in the blocking mixture, was added for 1 hr at RT. Finally, the cells nuclei were marked with Vectashield + DAPI. The sections were observed and captured with the optical microscope (Leica DM 5000 B + CTR 5000) with UV lamp and saved with IM 1000 software. Densitometric analysis of PCNA immunofluorescence was performed with ImageJ Software counting 20 seminiferous tubules/animal for a total of 100 tubules per group.

### 2.9. Statistical Analysis

Data were reported as mean ± standard error (SEM). Differences between the groups were considered statistically significant at *p* < 0.05. Analyses were performed using one-way ANOVA, Tukey’s post hoc t-test was applied when appropriate with Prism 5.0, GraphPad Software (San Diego, CA, USA).

## 3. Results and Discussion

**Figure 1:**
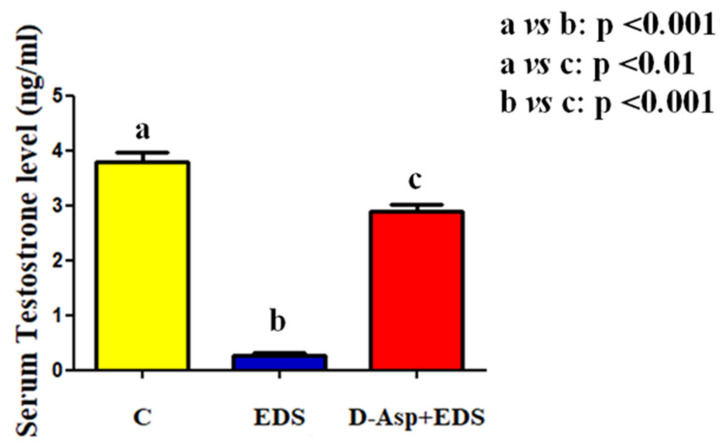
Serum testosterone (ng/mL) level from control, ethane dimethane sulfonate (EDS), and D-Aspartic acid (D-Asp) + EDS treated rats. In the EDS-treated rats, testosterone concentration strongly decreased, while in D-Asp + EDS treated animals, the decrease is less pronounced. Values are expressed as mean ± SEM from 5 animals in each group.

In this paper, we evaluated whether the use of D-Asp may have a protective role in preventing the testicular injury produced by the exposure to EDS, a toxicant that selectively destroys LC. Indeed, it must be considered that recently, some pollutants, such as cadmium [41,42,43] and BPA [44,45,46] may act as endocrine disrupters and/or toxicants that specifically affect gametogenesis leading to the production of low-quality gametes and, consequently, giving rise to a low rate of reproduction [47,48]. Firstly, to evaluate the effectiveness of the treatments, we measured the serum T levels. As shown in Figure 1, EDS injection caused a drastic drop in T concentration as compared to the control (*p* < 0.001), while a less pronounced decrease was evident in the serum of rats pre-treated with D-Asp (*p* < 0.01).

**Figure 2:**
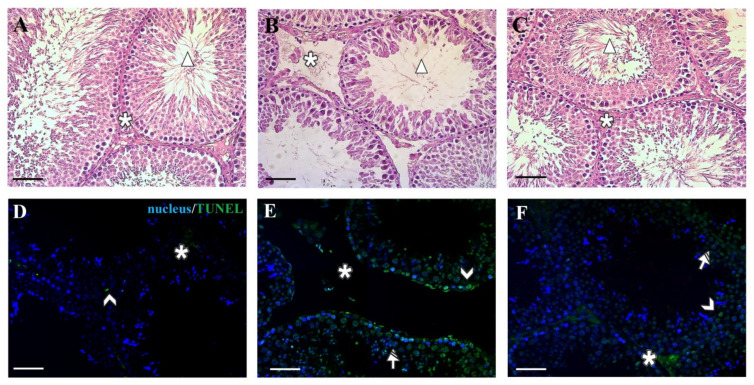
Hematoxylin-eosin and TUNEL staining of rat testicular paraffin-embedded sections. (**A**–**C**): Hematoxylin-eosin staining of control (**A**), EDS (**B**), and D-Asp + EDS (**C**) treated rat testes. The absence of Leydig cells in the EDS-treated group is evident; (**D**,**E**): Determination of apoptotic cells through the detection of TUNEL-positive cells (green) in control (**D**), EDS (**E**), and D-Asp + EDS (**F**) treated rats. Slides were counterstained with DAPI-fluorescent nuclear staining (blue). The images were captured at ×20 magnification. Scale bars represent 20 μm. Asterisks: Leydig cells. Triangles: luminal Spermatozoa. Arrowheads: Spermatogonia; Striped Arrows: Spermatocytes.

The observed androgen levels variation due to D-Asp and/or EDS treatment probably is responsible of the altered testicular histopathology, as shown in Figure 2. 

The control rats presented a normal interstitial compartment with many LC (asterisk, Figure 2A), with a regular seminiferous epithelium that is characterized by the presence of the germ cells in all the different stages of differentiation and the tubular lumen occupied by mature spermatozoa (triangle, Figure 2A). On the contrary, in the testis of rat that received EDS-injection, the absence of LC is noticeably evident (asterisk, Figure 2B), as a confirmation of its action on these cells. Moreover, an altered organization of the epithelium, as indicated by the loss of contact and the presence of empty spaces between the cells may be observed. It must be highlighted that in the lumen of many tubules, it was possible to observe the lack of spermatozoa (triangle, Figure 2B). In the D-Asp + EDS treated rats, it was appreciable a picture of the histological features that was no dissimilar to that of the control (Figure 2C). In fact, LC in the interstitial compartment were observed (asterisk, Figure 2C) and the organization of the adjacent germinal compartment was quite like that observed in the control testis. The above data were confirmed by the analysis of three different morphometric parameters, which showed that the epithelium thickness and diameter, as well as the percentage of tubular lumen occupied by SPZ were significantly lower in EDS-treated group as compared to the controls (Table 1). These results not only confirmed, once again, that EDS deadly action is specific for the interstitial LC, affecting the normal spermatogenesis, but also that the pre-treatment with D-Asp ameliorated its harmful effects on the hormonal and histological status.

The altered organization of the seminiferous epithelium observed in the testis of EDS-treated rats may be the direct consequence of T withdrawal, since it is well known that this hormone regulates the whole spermatogenetic process acting also as a “cell survival factor” to protect germ cells from apoptosis. Indeed, in the testis of control rats very few positive-TUNEL cells could be seen (Figure 2D). On the contrary, in the gonads of the EDS-treated animals, the few that left LC, still present in the interstitial compartment, were TUNEL-positive (asterisk, Figure 2E) as well as many spermatogonia (arrowhead, Figure 2E) and spermatocytes (striped arrow, Figure 2E) located in the adjacent germinal compartment. The fact that the increased number of TUNEL-positive cells may be a direct consequence of the T lacking is well supported by the D-Asp treatment, since in the testis of this group, this analyzed parameter is almost comparable to that observed in the control (Figure 2F), where the apoptotic signal was evident just in the nuclei of few LC (asterisk, Figure 2F), spermatogonia (arrowhead, Figure 2F), and spermatocytes (striped arrow, Figure 2F). Thus, the protective role exerted by D-Asp is evident not only in the maintaining the number of LC but also in preserving the appropriate organization of the germinal tubules, supporting the hypothesis that T is effectively required for germ cell survival and proliferation.

As a further analysis on the effects of D-Asp and/or EDS treatment on interstitial LC together with the testicular function, two LC “markers” have been evaluated: StAR protein and PREP.

**Figure 3:**
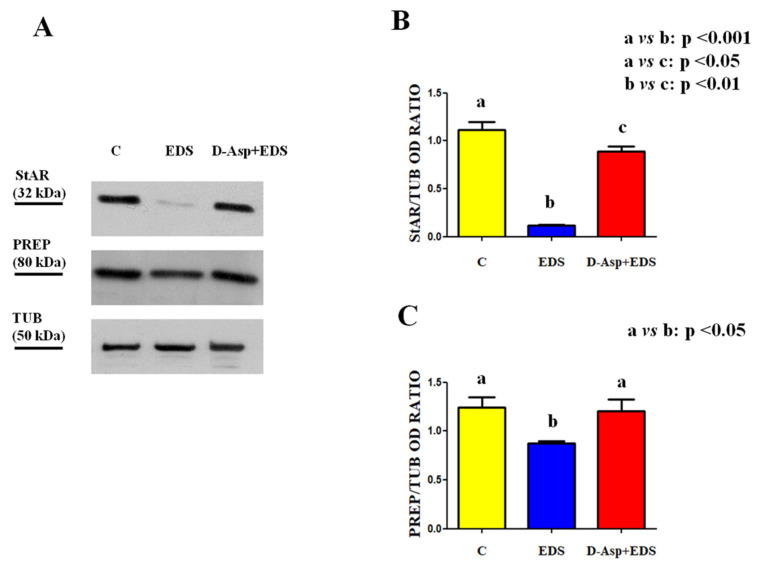
Testicular steroidogenic acute regulatory (StAR) and prolyl endopeptidase (PREP) protein levels in EDS and D-Asp + EDS treated rats. (**A**) Western blot analysis of StAR (32 kDa) and PREP (80 kDa) protein levels in the testis from control, EDS, and D-Asp + EDS treated rats. (**B**) The amount of StAR was quantified using ImageJ program and normalized with respect to α-tubulin (50 kDa). (**C**) The amount of PREP was quantified using ImageJ program and normalized with respect to α-tubulin (50 kDa). Values are expressed as mean ± SEM from 5 animals in each group.

Western blot analysis showed that the protein levels either of StAR or PREP are affected by EDS-treatment (Figure 3).

In particular, it was not a surprise to have found a drastic reduction of the StAR level as compared to control (*p* < 0.001; Figure 3A,B) while, interestingly, concerning PREP, its variation was less pronounced (*p* < 0.05; Figure 3A,C). This last point could be due probably by the fact that while StAR is specifically expressed by LC, PREP is widely distributed also in the germinal compartment, where its expression was unaffected by EDS, as discussed afterwards. In the rats that received D-Asp, prior the EDS injection, StAR level decrease was less marked (*p* < 0.01; Figure 3A,B) as compared to that observed in the testis of control animals. Similarly, PREP level in the D-Asp + EDS group was comparable to that of the control. These results support the hypothesis that D-Asp, in a certain manner, may prevent the drastic decrease in the LC number.

**Figure 4:**
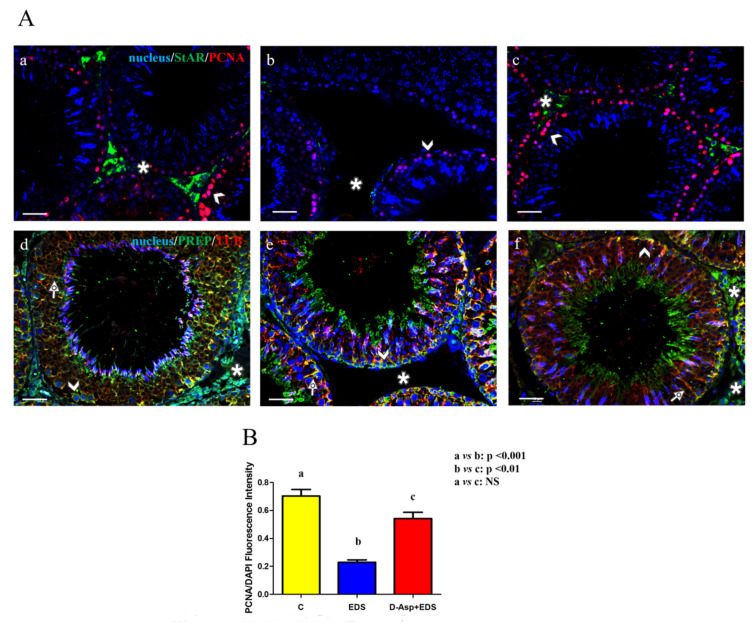
(**A**) StAR-PCNA and PREP-TUB co-localization in the testis from controls, EDS, and D-Asp + EDS treated rats. a-c: StAR (green) and PCNA (red) immunolocalization in the testis of control (**a**), EDS (**b**) and D-Asp + EDS (**c**) treated rats. d-e: PREP (green) and tubulin (red) immunolocalization in the testis of control (**d**), EDS (**e**), and D-Asp + EDS (**f**) treated rats. The yellow-orange intermediate tint indicates the two proteins co-localization. Slides were counterstained with DAPI-fluorescent nuclear staining (blue). The images were captured at ×20 magnification. Scale bars represent 20 μm. Asterisks: Leydig cells. Arrowheads: Spermatogonia; Arrows: Sertoli cells cytoplasm. (**B**) Histogram showing the quantification of PCNA fluorescence signal intensity using ImageJ. Values are expressed as means ± SEM from 5 animals in each group.

All the above results were strongly supported by immunofluorescence studies (Figure 4A) that revealed an altered signal of both StAR (Figure 4B) and PREP (Figure 4E) in EDS-treated testes.

In particular, we observed a specific localization of StAR in the interstitial LC (asterisk, Figure 4A(a)), while it was completely absent in the testis of the EDS-treated group, due to lack of LC (asterisk, Figure 4(Ab)). Moreover, the recovery of the StAR signal in the LC still present in the interstitial compartment of the D-Asp + EDS group was detected (asterisk, Figure 4(Ac)). We performed a labeling for PCNA, a commonly used proliferation marker, whose signal is prominent in those cells which dynamically replicate their DNA, such as spermatogonia (arrowhead, Figure 4(Aa)). Once again, the number of PCNA-positive cells is less accentuated in the EDS-treated animals (arrowhead, Figure 4(Ab)), while in the rat which also received D-Asp, an increased signal of PCNA was observable (Figure 4B). This could be due not only by the putative protective role exerted by D-Asp regarding EDS toxicity, but also by the fact that D-Asp itself is able to induce spermatogonia proliferation, activating MAPK pathway, as previously demonstrated by Santillo and colleagues [19].

PREP, together with tubulin, as highlighted by the intermediate yellow-orange tint, localized in all stages and, to varying extent, in all cell types; however, its immunopositivity was prevalently localized within the spermatogonia (arrowhead, Figure 4A(d)), Sertoli cell protruded cytoplasm (arrow, Figure 4(Ad)), and LC (asterisk, Figure 4(Ad)). In the testis sections of EDS-treated animals, we observed the absence of staining in the interstitial compartment (arrowhead, Figure 4(Ae)), together with a normal distribution in the germinal compartment. Interestingly, PREP immunofluorescence staining in rat testis sections from the D-Asp + EDS group was not different from that detected in the control group (Figure 4(Af)). This result is in accordance with that obtained by Western blot analysis, since the moderate decrease evidenced in the PREP protein level was attributable just to the absence of interstitial LC and it was not due to the deregulation of its expression in the cells composing the seminiferous epithelium.

**Figure 5:**
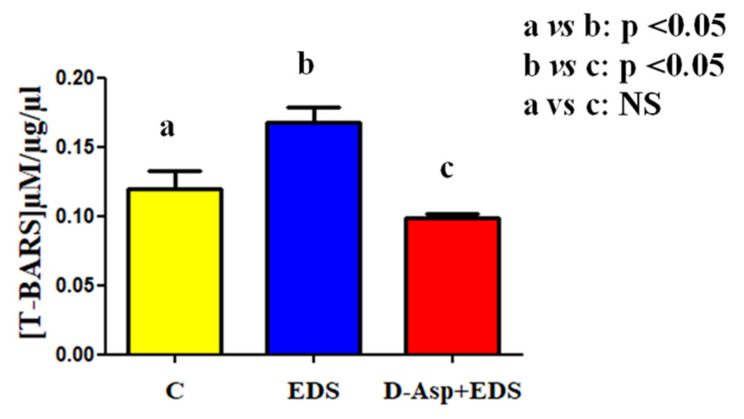
Lipid peroxidation evaluated by thiobarbituric acid-reactive species (TBARS) assay. Oxidative stress status evaluated via TBARS assay in testicular samples from control, EDS, and D-Asp + EDS treated rats. Values are expressed as means ± SEM from 5 animals in each group.

Although, taken together, all these data led us to hypothesize a possible role exerted by D-Asp in preventing EDS toxic action on LC, on the other hand, we had no information concerning the relative mechanism of action. We can speculate that a transesterification reaction between aspartic acid and EDS occurs, in which the corresponding aspartate ester is formed, and the alkylating effect of EDS would be neutralized. Many efforts have been addressed in this direction and among them, one of the most intriguing mechanisms implicated is the oxidative stress induced by EDS treatment. In fact, Zini and Schlegel [49] found that testicular lipid peroxidation significantly increased after EDS injection, together with a decreased catalase and phospholipid hydroperoxide glutathione peroxidase mRNA concentration [49]. In the present study, a similar result was obtained, since in the EDS-treated group, was observed a significant increase of the toxic reactive aldehydes, measured with thiobarbituric acid reactive substances (TBARS) assay (*p* < 0.05; Figure 5).

Worth remembering, TBARS are a byproduct of lipid peroxidation, thus it is an index of cellular oxidation status. It is interesting to remark that the co-treatment with D-Asp maintained the normal TBARS parameter, as compared to that of the control (Figure 5). This last data confirmed that one of the cytotoxic effects of EDS on LC may be mediated by the induction of oxidative stress, highlighted by the increased TBARS levels. Since no significant differences were observed between the control and D-Asp + EDS groups concerning this parameter, we could hypothesize that the D-Asp pre-treatment was effectively efficient in protecting testis just against the oxidative stress induced by EDS. This last hypothesis is also supported by several papers exploring the antioxidant properties of D-Asp. Many researchers demonstrated that D-Asp decreased oxidative stress and ameliorated sperm quality [27,50,51], protecting human spermatozoa by the increase of DNA fragmentation and lipid peroxidation in oligoasthenospermic men [52]. Therefore, it is possible to imagine a similar, antioxidant action exerted by D-Asp on EDS-induced toxicity in the rat testis, protecting LC from apoptosis and, consequently, maintaining a normal testicular environment.

## 4. Conclusions

To our knowledge, this is the first report evaluating the plausible role exerted by D-Asp in protecting/preventing EDS toxic action in the rat testis, ameliorating some analyzed factors, such as serum testosterone level, apoptotic rate, testicular StAR, and PREP protein levels and localization. Moreover, an oxidative stress parameter, the TBARS concentration, indicated that one of the possible mechanisms involved in this scenario is effectively the stimulation of oxidative stress. Although these findings undoubtedly require further investigation, we could safely hypothesize that D-Asp may be used as a useful tool to prevent and/or counteract the toxicity caused by the exposure also to other environmental pollutants, that act as endocrine disrupters, above all to preserve the production of good-quality gametes, to ensure an appropriate reproductive function.

## Figures and Tables

**Table 1:** Morphometric parameters evaluated in the rat testis.

Groups	Epithelium Thickness (µm)	Epithelium Diameter (µm) Empty Lumen (%)
C	80.94 ± 3.37a	237.61 ± 2.11a 27 ± 8a
EDS	51.16 ± 2.39b	179.81 ± 4.58c 69 ± 7d
D-Asp + EDS	69.91 ± 5.21c	221.04 ± 4.02a 33 ± 12a

Evaluation of histological parameters in EDS and D-Asp + EDS treated rats. Values are expressed as mean ± SEM from 5 animals in each group. a vs. b: *p* < 0.01; a vs. c: *p* < 0.05; a vs. d: *p* < 0.001, b vs. c: *p* < 0.05, a vs. a: NS.
